# Exploring Risk Factors and Patterns in Uncommon Recurrences of Varicella-Zoster Reactivation: A Review of Case Reports

**DOI:** 10.7759/cureus.83293

**Published:** 2025-05-01

**Authors:** Kiarra Krulikowski, Brittany Shectman, Dania Ilyas, Suzanne I Riskin

**Affiliations:** 1 Department of Foundational Sciences, Dr. Kiran C. Patel College of Osteopathic Medicine, Nova Southeastern University, Clearwater, USA

**Keywords:** atypical presentation, herpes zoster, immunocompromised, post herpetic neuralgia, reactivation, risk factors, shingles, varicella reactivation, vesicular rash, vzv vaccination

## Abstract

Varicella-zoster virus (VZV) causes chickenpox and then establishes latency in the autonomic ganglia. Reactivation of the virus, known as herpes zoster or shingles, manifests as a unilateral, vesicular rash localized within one dermatome accompanied by pain and pruritus. While the classic rash resolves within three weeks, older or immunocompromised individuals may experience prolonged symptoms, increased vesicle number, and complications such as post-herpetic neuralgia. Although the classic manifestations of VZV are well known, more cases are appearing with an atypical presentation. We highlight eight reports of unusual presentations describing confirmed cases of VZV that become reactivated and note a wide range of ages, with half over 70 years of age and half under 40 years of age, two including children. Unusual presentations include zoster sine herpete, vocal fold paralysis due to vagal nerve involvement, and encephalitis with massive pulmonary emboli in a previously healthy 37-year-old woman. One case features a child who developed shingles from the vaccine strain of VZV. Diagnostic delays occurred in all cases due to the atypical nature of the presentations, often resulting in initial misdiagnoses and inappropriate treatments such as antibiotics or corticosteroids. Despite eventual antiviral therapy, two patients experienced incomplete recovery, suffering from persistent neuropathic pain or muscle atrophy. These cases emphasize the variability in VZV presentations, the importance of timely diagnosis, and the need for greater clinical awareness to prevent delayed treatment and adverse outcomes.

## Introduction and background

Varicella-zoster virus (VZV) is a member of the *Herpesviridae* class of viruses, embodies a double-stranded DNA genome, and becomes latent in the human body similar to other herpesviruses [1]. Initial infection with VZV yields an outbreak of chickenpox, also known as varicella, while a reactivation of VZV most commonly yields shingles/herpes zoster virus (HZV). Following the initial infection, VZV remains latent in various locations, including dorsal root ganglia, cranial nerve ganglia, autonomic ganglia in the enteric nervous system, and astrocytes [2]. When reactivation occurs, the VZV replicates in the cell bodies of the neurons until particles of the virus shed down the nerve to the respective dermatome and cause inflammation along with the classic herpetic rash described as vesicles on an erythematous base [2]. The vesiculation of HZV typically appears in a unilateral fashion along one dermatome and is accompanied by pain, pruritus, or both. The typical course of infection, including the resolution of the rash, heals quickly within one week and does not leave any scarring in immunocompetent individuals. The rash, as well as systemic symptoms, can become severe and last for weeks or longer in the elderly and immunocompromised [3]. Atypical cases can present as neurological or vascular symptoms, sometimes not including a rash at all. Differences in clinical symptoms do not seem to be related to detectable viral load, according to one study [4]. Quinlivan et al. found that viral DNA was detected in 101 out of 130 patients with acute zoster and following a univariate analysis with the remaining 101 patients, it showed that a higher viral load was only significantly associated with the presence of prodromal symptoms and no association with gender, immune status, rash age, or severity of the pain [4].

The most well-known complication following a case of HZV is post-herpetic neuralgia (PHN). This has been noted in especially severe cases of zoster in which the patients had a large number of vesicles as well as intense pain. PHN typically appears within the three months following the resolution of the zoster rash and is described as a burning, throbbing, or shooting pain [3]. The pain can range from mild to severe and last anywhere from a few months to a year or longer [3]. There is no cure, only symptomatic treatment.

Vaccines are available for both the prevention of varicella in children as well as the prevention of zoster in adults. The varicella vaccine was approved in 1996 for routine administration with the guidelines of one dose for children 1 to 12 years of age and two doses of the vaccine for children 13 years of age and older [5]. These guidelines changed in 2005 when it was recognized that there was still a high occurrence of breakthrough infections. The new guidelines recommend two doses of the vaccine, with the first dose at 12-15 months of age and the second dose at four to six years of age [5]. The implementation of the varicella vaccine demonstrated a 90% decrease in incidence of the disease from 1995 to 2005, with a further decrease of 83.9% after the updated guidelines for vaccination in 2005. For prevention of herpes zoster and further complications, there are two vaccines currently available: a live attenuated VZV vaccine and a recombinant adjuvanted VZV vaccine that contains the glycoprotein subunit E [2]. While the live attenuated vaccine has been customary for years, the recombinant adjuvant vaccine has proven more efficacious and is safer for use in immunocompromised children and adults [2].

The reactivation of VZV causing HZV is dependent on any number of internal and external factors. The typical clinical manifestation of shingles, being prodromal neuropathy followed by unilateral pruritic vesiculation, is well known; however, there are more case reports becoming available that showcase atypical manifestations. In this review, we will discuss the uncommon presentations of HZV and the associated risk factors or patterns specific to the atypical reports.

This article was previously presented as a meeting poster at the 2025 American Academy of Dermatology (AAD) Annual Meeting on March 8, 2025.

## Review

Methods

**Figure 1:**
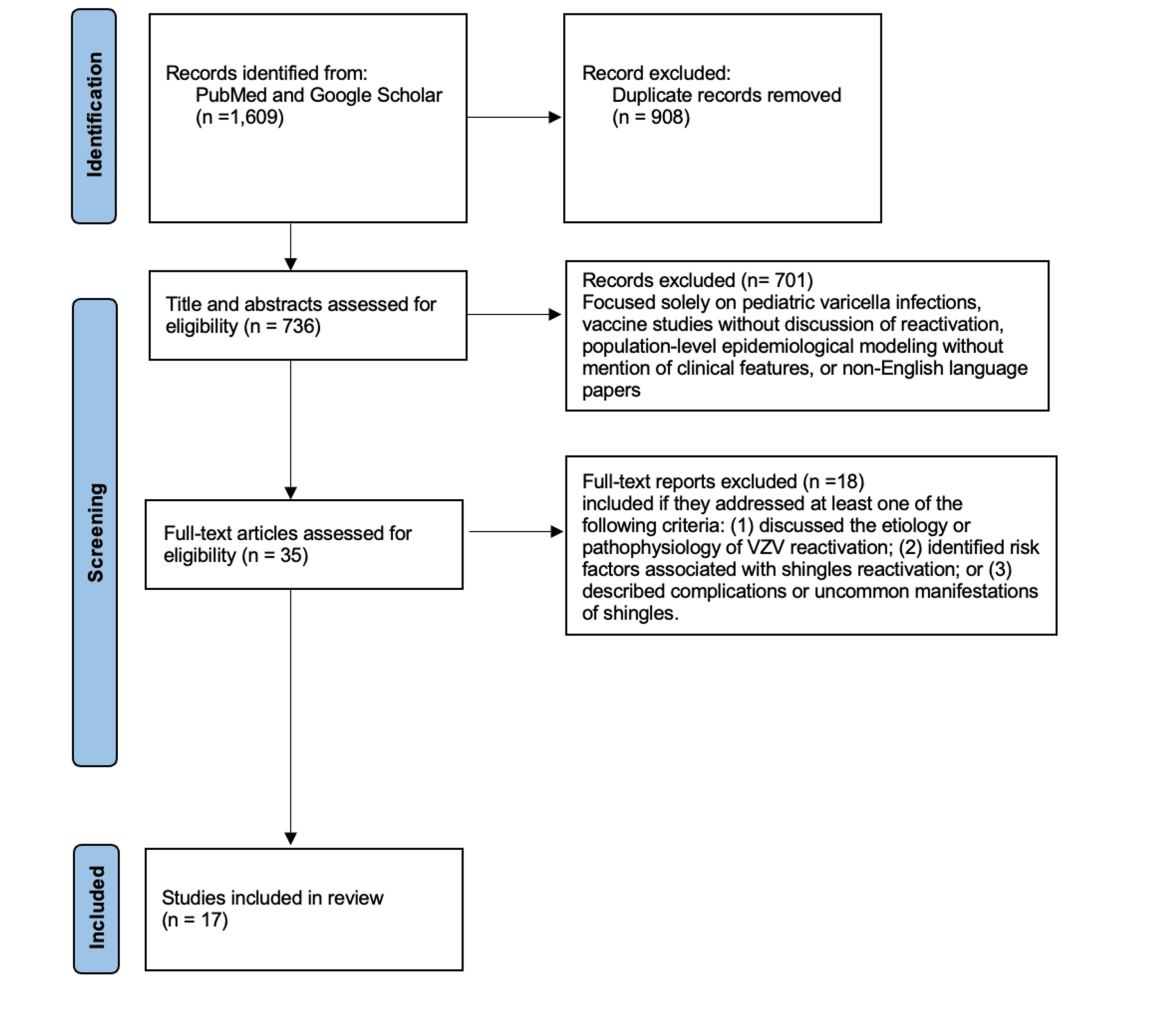
Preferred Reporting Items for Systematic Reviews and Meta-Analyses (PRISMA) flowchart VZV: varicella-zoster virus

The search string consisted of terms including "varicella" OR "shingles," combined with “risk factors,” “complications,” “uncommon,” and “reactivation” using Boolean operators (AND/OR). To ensure relevance and quality, only peer-reviewed articles published from 2014 onward were included. Searches were conducted using PubMed and Google Scholar, yielding a total of 1,609 articles. After removing duplicates, 736 articles remained. Title and abstract screening was conducted to identify studies explicitly discussing VZV or HZV in the context of the above-mentioned keywords. Articles were excluded if they focused solely on pediatric varicella infections, vaccine studies without discussion of reactivation, population-level epidemiological modeling without mention of clinical features, or non-English language papers. This screening led to the exclusion of 701 articles. A full-text review was then performed on the remaining 35 articles. Studies were included if they addressed at least one of the following criteria: (1) discussed the etiology or pathophysiology of VZV reactivation; (2) identified risk factors associated with shingles reactivation; or (3) described complications or uncommon manifestations of shingles. As a result, 17 articles were included in the final review. Among these, eight were case reports detailing rare presentations of VZV reactivation. All articles were confirmed to be peer-reviewed by checking Ulrich’s database. Figure 1 is a Preferred Reporting Items for Systematic Reviews and Meta-Analyses (PRISMA) flow diagram highlighting our search and review process.

Review

Risk Factors, Atypical Presentations, and Complications

HZV has been linked to numerous chronic factors that predispose people to an earlier onset. Some risk factors are more serious than others, but they all lead to an increased risk of experiencing HZV. Risk factors can range from unavoidable factors, such as increasing age, to other conditions of immunocompromised states. According to a meta-analysis done by Steinmann et al. [6], patients under immunosuppressive therapies were the most likely to develop HZV. Due to VZV's tendency to remain dormant in the sensory ganglia as a result of cell-mediated immunity, older patients and immunocompromised individuals are at a much higher risk. The peak age of documented HZV is in the age group of 60-69, with an increasing incidence of hospitalization in patients over 72 [7]. Risk factors can also be exacerbated by the timing of initial treatment. If treatment of HZV is provided within 72 hours of rash onset, it is more likely that the severity of progression will be decreased [7]. Although gender is still being studied as a possible risk factor, women tend to be more likely to progress from dormant VZV to HZV [6]. A major side effect of HZV is PHN, a continuous nerve pain in the area of skin affected by shingles. Although the risk factors are similar to HZV in general, there tends to be a discrepancy in incidence and age. There is an increased risk of PHN in patients over 80 years old or younger than 50 [7]. Diseases related to lifestyle habits, such as cardiovascular disease and diabetes, have also been linked to developing herpes zoster, with an increased exacerbation of hospitalization [8]. Recent studies have linked these risk factors to increased instances of prolonged pain, stroke, and severe cardiovascular disease [8]. Understanding these risk factors and getting vaccinated for VZV is crucial to mitigate the long-term effects of contracting VZV.

VZV reactivation comes with an array of neurological complications - PHN being the most frequent finding. PHN is a prolonged manifestation of shingles where the rash subsides, but pain remains. This complication has the potential to elicit a substantial amount of pain in affected individuals. Unfortunately, current treatment options only provide minimal relief due to the causative agent of PHN being unidentified [9]. VZV reactivation can also result in a spectrum of vasculopathies, most often identified by the findings of VZV-specific antigens and VZV DNA in cerebral arteries [8]. Of the vasculopathies produced by VZV reactivation, including cerebral aneurysms and spinal cord infarction, the clinical presentations can range from declining function to seizures [9]. Giant cell arteritis, which produces inflammation in temporal arteries, causing subsequent irreversible blindness, is yet another suspected rare complication of VZV reactivation [9]. VZV meningoencephalitis, which presents with usually mild symptoms of fever, confusion, headache, and meningeal symptoms, has been detected by polymerase chain reaction (PCR), showing a positive correlation for VZV [9]. Segmental motor weakness is another complication of zoster, where onset typically presents within two weeks, with varying intervals. These motor weaknesses can extend to the diaphragm and intercostal muscles, making it an even more serious side effect of VZV [9]. Diagnosis in these cases is established both clinically and with PCR detection of VZV DNA in cerebrospinal fluid [9]. In some cases, neurological complications elicited by VZV reactivation can be seen without the presence of the usual rash, known as zoster sine herpete [9]. These cases are confirmed through VZV DNA in CSF, as well as the presence of CSF anti-VZV IgG antibody [9]. Reactivation of VZV can produce ophthalmic complications such as retinitis, resulting from its involvement with the trigeminal nerve [9]. When VZV reactivation affects the facial nerve, it can cause Ramsay-Hunt syndrome, which presents with facial palsy, severe ear pain, and a vesicular rash [9]. Another neurological manifestation of VZV reactivation is Guillain-Barre syndrome (GBS), which presents as weakness or tingling that spreads to the arms and face, usually ending with paralysis [9]. VZV has also been implicated in the development of dementia and an increased risk of Alzheimer’s disease, with antiviral therapies linked to a decreased risk of dementia [8]. Additionally, progressive multifocal leukoencephalopathy was described in two patients previously infected with zoster in what can be viewed as the opportunistic infection of VZV directly into brain cells [9].

Case Report Series

Case report 1: This case highlights a nine-year-old boy in British Columbia who presents to a clinic during the summer months with a rash of one day, accompanied by a stinging pain. The rash itself is described as patchy, flat, non-blistering, eczematous, and covering the dermatomes of C6, C7, and C8 [10]. A skin specimen was taken and ultimately revealed VZV, determined by PCR, and further examination of this strain showed that it was the vaccine strain of the virus. Oral acyclovir was prescribed, and within three weeks, the rash healed completely. The boy did not have a history of chickenpox, but was vaccinated for VZV before entering kindergarten [10]. A common side effect of the VZV vaccine can include a mild eruption at the injection site, but a late reactivation of the virus is considered exceedingly rare, although it has been reported [10]. An American study looked at the incidence rates of zoster eruption between vaccinated and unvaccinated children and found that the rates were about 48 per 100,000 and 230 per 100,000, respectively [10]. It was further concluded that in the vaccinated group, over half of the cases were associated with the vaccine strain of the virus, and the remainder were the wild-type strain. This suggests that even vaccinated children may become infected by the wild-type virus and eventually develop zoster years later [10]. Both strains, wild type and vaccine strain, tend to present almost identically.

Case report 2: Although rare, VZV reactivation can occur without the expression of vesicular rash in young immunocompetent individuals. One such incident involved a 12-year-old girl who presented with cough, rhinitis, fever, and complaints of a worsening frontal headache six days prior to admission [11]. Her medical history was unremarkable aside from infantile chickenpox. Upon admission, the patient did not show any signs of neurological deficit, her vital signs were stable, and the rest of her clinical examination was unremarkable. Antibiotic therapy was initiated on the second hospital day following nasal endoscopy findings consistent with acute bacterial sinusitis [11]. The next day, the patient’s headache worsened, accompanied by altered mental status (AMS), psychomotor agitation, reduced responsiveness to stimuli, and drowsiness. Examination of the patient's CSF fluid demonstrated lymphocytic pleocytosis, PCR detection of VZV, and serological testing showing IgG antibodies against VZV (not accompanied by IgM against VZV), confirming VZV reactivation in the patient. The patient’s treatment consisted of antiviral therapy with acyclovir, rapidly improving her clinical condition the next day, with continued improvement of her symptoms throughout the course of her treatment [11].

Case report 3: A 30-year-old male made a visit to his primary care provider due to complaints of low-grade fever, general malaise, and a sore throat. He was diagnosed with an upper respiratory tract viral infection and was treated with ibuprofen and paracetamol. The next day, a rash developed on his left anterolateral chest that extended to his back on the same side and was described as erythematous, pruritic, and raised [12]. The patient then presented to the emergency department (ED) 12 hours after noticing the rash and was diagnosed as having an allergic reaction to the treatment provided by his primary care provider. Treatment in the ED consisted of IV antihistamine and hydrocortisone, and was discharged with topicals of the same two medications. Another 12 hours passed, and the patient visited a different ED complaining of worsening symptoms. Examination showed he was febrile, tachycardic, had exudate on his tonsils and cervical lymphadenopathy [12]. After testing, it was revealed he was positive for influenza B, and he was treated in the hospital and discharged with treatment to continue at home. The patient waits another day before visiting the ED again, where he was referred to dermatology on the same day. A biopsy was performed by the dermatologist, and in the meantime, he was prescribed clobetasol 0.05% topical cream and oxycodone oral tablets [12]. He followed up with the dermatologist two weeks later and reported that the rash yielded raised vesicles that turned into a cluster of white, crusted erosion surrounded by an area of patchy erythema. The biopsy that was taken two weeks prior revealed a positive result of herpes zoster, and was then prescribed oral valacyclovir for a week [12]. The official diagnosis became multidermatomal herpes zoster, which is a rare manifestation of the virus, as this only occurs in roughly 16% of cases, but it is typically seen in the immunocompromised, which this patient was not.

Case report 4: In one such report, a 37-year-old female presented to the emergency room with the onset of fever, refusal to eat or drink, and psychomotor impairment [13]. Upon admission, an increase in C-reactive protein, abnormal liver function, and prerenal acute kidney injury were detected. The patient was found to have a zoster sine herpete per serology with concurrent inferior vena cava (IVC) thrombosis and massive bilateral pulmonary emboli found with CT imaging [13]. The neurologic manifestations were attributed to a possible case of encephalitis. Antithrombotic therapy with enoxaparin was immediately started, along with ceftriaxone and IV acyclovir empirically. The acyclovir was continued over a three-week period along with antithrombotic treatment. Risk factors for the patient were minimal, making this case even more atypical; the patient had no history of immunocompromised state or previous history of coagulopathy. After examining the laboratory and radiological exams, VZV was deemed to be the cause of not only the acute hepatitis but also the thromboembolic sequelae.

Case report 5: Pertinent to this case is a 70-year-old male who presented to the ED with neck pain on his right side and lymphadenopathy of the submandibular region. His history was positive for invasive anal squamous cell carcinoma that was treated by excision and chemotherapy [14]. This patient goes on to progress with dysfunction of cranial nerves X and XI, which present as dysphonia, dysphagia, and atrophy of the trapezius and scalene muscles. It was concluded via antibody titer screening that all of the patient’s symptoms were due to a consequence of varicella-zoster [14]. Treatment included injections into the patient’s vocal cords with Botox, which alleviated the dystonia and dysphagia. He was also given oral acyclovir and valacyclovir, but because of the delay in diagnosing and treating, the neuropathic pain as well as muscle atrophy remained [14].

Case report 6: A 74-year-old woman had a history of a periorbital vesicular rash as well as some eye redness that began two weeks ago, with a change of blurred vision in her left eye that started three days ago. She was given oral acyclovir as well as ciprofloxacin a week before the current presentation [15]. Examination findings of the left eye consisted of edematous eyelid, diffuse injection of the conjunctiva, and corneal stromal edema. Further investigation revealed a streak of hypopyon with a hyphema level below in the anterior chamber [15]. Based on these findings, the diagnosis was ruled to be herpes zoster ophthalmicus (HZO) with keratouveitis. Treatment was started and included acyclovir eye ointment, eye lubricants, ciprofloxacin eye drops with dexamethasone [15]. After one week of treatment, there was improvement in the hyphema level, and the hypopyon was healed. The patient did eventually develop raised intraocular pressure, so she was started on an antiglaucoma medication. Two weeks post-starting the new treatment, an epithelial defect was seen but resolved about a month into treatment. Around three months of follow-up, the epithelial defect had returned along with hypopyon hyphema level [15]. With the epithelial defect and corneal edema persisting, the recommendation was surgical intervention; however, the patient wanted to stick with the conservative route.

Case report 7: In one case involving a 79-year-old male with cholangiocarcinoma who presented with abdominal pain and a widespread papulovesicular rash, initially presumed to be a drug eruption, was eventually diagnosed as VZV [16]. The patient had been taking capecitabine for a few weeks prior to the development of a rash on his face, chest, and back, which, combined with preliminary biopsy findings, prompted clinical suspicion of a drug eruption. Capecitabine usage was stopped, and steroid therapy was initiated [16]. Further dermatological evaluation and biopsies of the lesion revealed cytopathic effects consistent with VZV infection, which prompted discontinuation of steroids and implementation of airborne precautions. PCR detection of VZV prompted treatment with IV acyclovir, and the patient was discharged with valacyclovir for seven days with subsequent resolution of the rash within a few weeks [16].

Case report 8: Zoster sine herpete is a very rare form of VZV in which patients present with radicular pain without rash. Herpes zoster spreading may manifest as multiple signs and symptoms due to various combinations of cranial neuropathies [17]. It is uncommon to see vagus nerve symptoms without cutaneous lesions, but in this report, an 80-year-old man presented in this fashion. The patient presented to otolaryngology with sore throat, dysphagia, and hoarseness [17]. Upon endoscopic examination, unilateral vocal fold paralysis was and subsequent PCR testing confirmed VZV. The patient was treated with valacyclovir and corticosteroids, which thankfully led to complete recovery within two months. This unusual presentation of an isolated neuropathy is what made the case so difficult to diagnose. Table 1 highlights the main points from each case.

**Table 1:** Summary of case report highlights

Case Number	Author	Age, Gender	Risk Factors	Clinical Features	Complications	Treatment
1	Cimolai et al., 2014 [10]	9, M	Varicella-zoster virus vaccination	Rash	N/A	Acyclovir
2	Ciancia et al., 2020 [11]	12, F	N/A	Rhinitis, fever, cough, frontal headache, altered mental status	Varicella-zoster virus encephalopathy	Antibiotic followed by acyclovir
3	Alhayyas et al., 2020 [12]	30, M	N/A	Fever, sore throat, rash	Multidermatomal Herpes zoster	Ibuprofen, paracetamol, antihistamine, hydrocortisone, dobetasol, valacyclovir
4	Salvotti et al., 2023 [13]	37, F	N/A	Fever, psychomotor impairment	Inferior vena cava thrombosis, bilateral pulmonary emboli	Enoxaparin, ceftriaxone, acyclovir
5	Wang et al., 2021 [14]	70, M	Age	Neck pain, submandibular, lymphadenopathy, dysphonia, dysphagia	Neuropathic pain, muscle atrophy	Botulinum toxin, acyclovir, valacyclovir
6	Katherine et al., 2020 [15]	74, F	Age	Periorbital rash, diplopia	Epithelial defect, corneal edema	Acyclovir, ciprofloxacin
7	Adimora-Onwuka and Hall, 2022 [16]	79, M	Age	Abdominal pain, papulovesicular rash	N/A	Steroids, intravenous acyclovir, valacyclovir
8	Hosseini et al., 2015 [17]	80, M	Age	Sore throat, dysphagia, vocal cord paralysis	N/A	Valacyclovir, corticosteroids

Discussion

The goal of reviewing this series of cases was to try to determine if there were any patterns between patient characteristics, treatment, or outcomes. The patient population consisted of two children, two patients in their mid-30s, and four patients aged 70 or older. All eight of the patients were immunocompetent at the time of presentation; however, the older age of half the patients may be a key factor in why those four developed a more severe and atypical case of herpes zoster. The younger half of the patients represent the most interesting out of all the cases, such that their history implies they were in good health and not in a risk factor group for age. Vaccination or prior illness status was only revealed in the two cases regarding children, where the nine-year-old male was previously vaccinated and the 12-year-old female had a case of infantile chickenpox. A common theme between all cases was a delay in appropriate treatment for herpes zoster, which was due to the atypical presentation at the time they were seen. Most patients were given a prophylactic treatment consisting of antibiotics, analgesics, or corticosteroids before eventually receiving either acyclovir or valacyclovir. With the correct diagnosis and treatment, six cases fully recovered, but two cases had lasting effects from the virus due to the delay of treatment and the request for conservative measures.

## Conclusions

Even though shingles is a seemingly common condition to diagnose and treat, there is evidence to suggest that the incidence rates are higher due to the abnormal ways the virus may present itself. With a classic presentation, diagnostic tests may not even have to be performed, and a patient treated off clinical signs alone. However, we have seen through this case series that patients are presenting with cases of zoster that are unrecognizable and therefore have a delay in correct treatment, with a potential for worse patient outcomes. A thorough history taking is an essential first step in order to determine the risk factors for varicella reactivation, followed by expanded testing parameters. This review is limited by its exclusion of non-English language studies, which may omit relevant international findings. Restricting the search to articles published from 2014 onward could overlook earlier, potentially valuable research. Additionally, reliance on only two databases (PubMed and Google Scholar) may limit the comprehensiveness of the literature search. This review could begin to pave the way for new protocols when it comes to diagnosing and treating patients with suspected shingles.
